# Breastfeeding practices among foreign-born non-Hispanic mothers of children in the United States – a cross-sectional study of nationwide multi-year data

**DOI:** 10.1186/s12884-026-09082-5

**Published:** 2026-04-17

**Authors:** Aishat Gambari, Shivani A. Patel, Melissa F. Young, Sarah C. Blake, Elizabeth C. Rhodes, Usha Ramakrishnan

**Affiliations:** 1 Doctoral Program in Nutrition and Health Sciences, Laney Graduate School, Emory University, 1518 Clifton Rd N E, Atlanta, Georgia 30322 USA; 2 Hubert Department of Global Health, Emory University, Atlanta, USA; 3 Department of Health Policy and Management, Emory University, Atlanta, USA

**Keywords:** Breastfeeding, Maternal & Child Health, Immigrant Health, Health Equity

## Abstract

**Background:**

Existing research has documented various predictors of immigrant breastfeeding, but the research and related interventions have mostly focused on Hispanic populations. Little is known about predictors of relative importance for successful breastfeeding among the growing population of foreign-born non-Hispanic (FBNH) mothers in the U.S. This study examines breastfeeding practices among FBNH mothers relative to other U.S. groups and explores socioeconomic factors associated with FBNH mothers’ ability to meet recommended feeding practices.

**Methods:**

Cross-sectional data on U.S. children 0–5 years from the 2022–2023 National Survey of Children’s Health were used to examine breastfeeding initiation, exclusive breastfeeding (EBF) for 6 months, breastfeeding for ≥ 12 months, and optimal breastfeeding (all 3 practices) among FBNH, foreign-born Hispanic (FBH) and U.S.-born mothers. Chi-square tests compared proportions and multivariable binomial regression models estimated adjusted prevalence ratios for breastfeeding outcomes by nativity & ethnicity, maternal employment status, community and social support, and Special Supplemental Nutrition Program for Women, Infants, and Children (WIC) participation.

**Results:**

Most FBNH mothers (87.7%) initiated breastfeeding, while about one-quarter (28.2%) practiced EBF for 6 months. More than half (52.9%) reported breastfeeding for at least 12 months and about one-quarter (24.1%) reported meeting all three practices. U.S.-born mothers had significantly lower prevalence of BFI (83.1% vs. 87.7%, *p* = 0.04), while FBH had significantly higher prevalence of EBF at 6 months (31% vs. 28.2%, *p* = 0.02) and overall optimal breastfeeding (28.8% vs. 24.1% *p* = 0.01) in the adjusted models, when compared to FBNH mothers. Maternal employment status, community and social support, and WIC participation were not associated with breastfeeding outcomes among FBNH mothers.

**Conclusion:**

Despite higher rates of breastfeeding initiation, only one in five FBNH women achieved all recommended breastfeeding practices, and over 70% did not meet exclusive breastfeeding recommendations. Further research should investigate key drivers of breastfeeding practices and identify factors of relative importance to guide culturally appropriate and context-specific programs targeting this population.

**Supplementary Information:**

The online version contains supplementary material available at 10.1186/s12884-026-09082-5.

## Background

Breastfeeding protects against many acute and chronic childhood conditions and promotes cognitive development in children [1–4]. Breastfeeding also reduces women’s risk of breast and ovarian cancers, type-2-diabetes and high blood pressure later in life [1, 5–12]. Given these benefits, the U.S. Department of Agriculture, World Health Organization and American Academy of Pediatrics recommend early breastfeeding initiation (within the first hour of birth), exclusive breastfeeding (EBF) for six months and sustained breastfeeding for one [13] or preferably two years [14, 15].

The U.S. has made great progress in improving national breastfeeding initiation rates but exclusive and sustained breastfeeding rates remain suboptimal [16]. According to the U.S. Centers for Disease Control’s 2025 Early Childhood Nutrition Report, 85% of children initiated breastfeeding but only 27.9% of infants were EBF at six months and 40.8% were breastfed for at least one year [17]. These national rates are far below Healthy People 2030 goals of 42.4% EBF and 54% sustained breastfeeding through 12 months [18], and many mothers stop breastfeeding earlier than planned [17]. Furthermore, there are complex inequities in breastfeeding outcomes by race, ethnicity, socioeconomic status and geographical location, but the drivers and nuances within these disparities remain unclear, especially among foreign-born populations [18–22].

Understanding breastfeeding among foreign-born populations is important because immigrants currently make up about 16% of the U.S. population [23, 24], and accounted for 65% of the total population increase from 2021 to 2022 [25]. Foreign-born mothers are reported to have high breastfeeding intention [26–29] and initiation [27, 29–31], but low EBF rates [27, 28, 30, 31]. Also, studies have reported that foreign-born women who EBF in their home countries tend not to do so when they migrate to the U.S [32–34], and their breastfeeding rates decrease the longer they reside in the U.S [35–39]. However, most of these studies were qualitative and included a predominantly Hispanic population. Challenges related to socioeconomic status, migration status, increasing acculturation, and lack of support were associated with decreased rates of EBF among immigrants [27, 40–44].

Most of the existing evidence on immigrant breastfeeding are based on mothers of Hispanic descent and less is known about breastfeeding outcomes of non-Hispanic foreign-born mothers [45], despite increasing immigration from various parts of Asia and Africa [46, 47]. Understanding the factors that influence optimal breastfeeding practices among the increasing population of foreign-born non-Hispanic mothers is important for achieving the Healthy People 2030 goals [18]. Also, most of the literature on immigrant breastfeeding focuses on breastfeeding beliefs and breastfeeding initiation and duration [39, 48] and information on EBF practice among immigrants is limited.

Using National Survey of Children’s Health (NSCH) data [49], this study examines breastfeeding among FBNH mothers by (i) describing and comparing their breastfeeding practices with foreign-born Hispanic and U.S.-born mothers, and (ii) exploring associations between 3 key socioeconomic factors – community and social support, WIC participation, and maternal employment status- with recommended breastfeeding practices among FBNH mothers of U.S. children.

## Methods

This cross-sectional study analyzed data on children 0–5 (*n* = 41256) from the combined 2022–2023 NSCH. NSCH is an annual nationally representative cross-sectional survey that collects data on the health, well-being, access to healthcare, demographic, and socioeconomic status of non-institutionalized children 0–17 years old and their caregivers. NSCH includes caregiver reported breastfeeding data for children 0–5 years [50, 51].

**Fig. 1:**
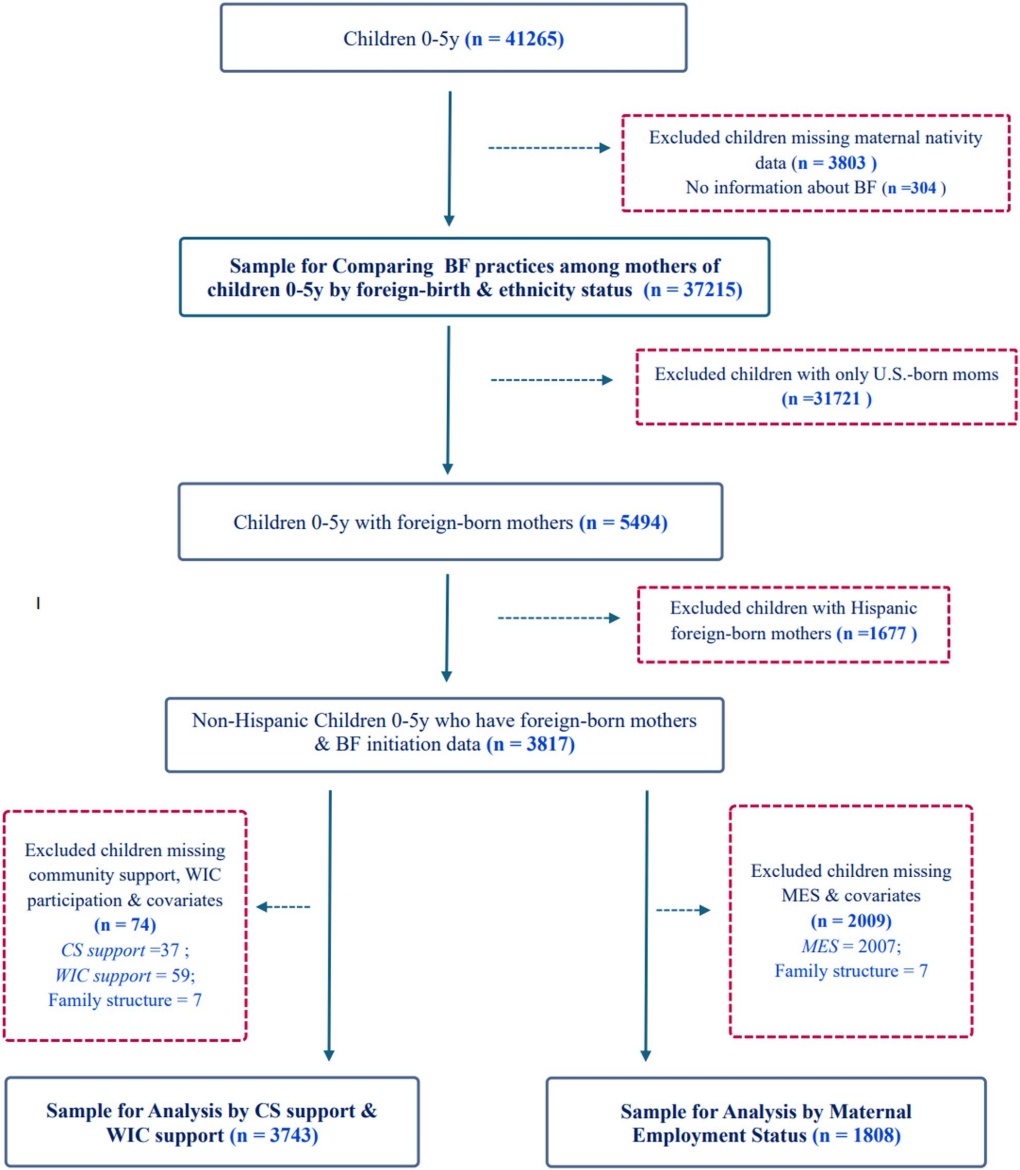
Flow diagram for determining the analytic samples Flow diagram for determining the final analytic sample sizes for comparison of breastfeeding practices by key socioeconomic variables – Community and social support, WIC participation and Maternal employment status

We classified mothers into three groups: (1) Foreign-born non-Hispanic (FBNH); (2) Foreign-born Hispanic (FBH); and (3) U.S.-born, based on nativity and ethnicity. Mother’s nativity was determined by identifying female parents that also reported being foreign-born. Parental relationship was determined from responses to questions about caregiver(s) relationship with the child. Foreign-born mothers were classified by ethnicity status to compare our main target population, FBNH, with the relatively well-studied FBH. We used the child’s Hispanic origin status as a proxy to classify mothers as non-Hispanic or Hispanic origin. Children who were missing data for any of the key variables were further excluded in a stepwise process (Fig. 1).

### Outcome variables

The outcomes of interest were breastfeeding initiation (BFI), exclusive breastfeeding for six months (EBF), duration of breastfeeding (BF duration) and overall optimal breastfeeding (all three practices), coded as binary variables. *BFI* was defined based on whether a child was reported as being ever breastfed or fed breastmilk. *EBF for 6 months* was based on children 0–5’s reported age at introduction of formula or other liquid and solids. Children for whom other foods or formula were introduced at 6 months or higher were considered to have been exclusively breastfed for 6 months; those who were less than 6 months old were coded as missing EBF data. *BF duration* was calculated based on each child’s age (months) at breastfeeding cessation and recoded as a binary variable using a cutoff of 12 months [13]. Since other major global and national health organizations recommend breastfeeding for the first 2 years of a child’s life, we calculated prevalence of BF for at least 24 months but used the 12-month cutoff for bivariate and multivariate analyses due to sample size. Children who were older than 12 months old and still breastfeeding at the time of survey were coded as having BF for longer than 12 months. *Optimal Breastfeeding* (optimal BF) is defined as a composite binary variable that combines all three recommended breastfeeding practices.

### Socioeconomic factors

This study explores 3 structural and moderately intervenable variables across interpersonal, policy, and individual levels – community and social support [52–54], WIC participation [32, 48, 55], and maternal employment status [9, 48, 56] – in relation to the breastfeeding outcomes. These variables were identified from a review of breastfeeding literature as important predictors of breastfeeding success [9, 18, 32, 38], and we wanted to see if this was true for the FBNH population as well. *Community and social support (CS support)* – children were assigned a score of 0–5 based on their caregiver’s responses to 5 questions about neighborhood, community and emotional support. These scores were then collapsed into 3 categories (0–1: little or no support; 2: moderate support, 3–5: high support). *WIC participation* was defined as a binary variable based on whether caregivers received WIC benefits within the past 12 months. *Maternal employment status (MES)* was coded as a 3-level categorical variable (full-time, part-time and unemployed) and explored only among a subset of children for whom maternal employment data was reported. Missing responses were compared to check for selection bias in those who reported MES data (see Additional File 1).

### Covariates

Covariates of interest were mother’s age, mother’s education level, race of child, presence of multiple young children (0–5) in household, household poverty level, and household structure. *Mothers’ age* was considered as a continuous variable and *mothers’ education level* was explored in 3 categories- high school graduate or less, some college/technical school, and college or higher. *Race of selected child* was classified into 5 categories- Hispanic, White, Black, Asian and Other/Multiracial categories, *presence of multiple children 0–5 in the household* was assessed as a binary variable and *household poverty level* was defined based on percent of poverty level and assessed in 4 categories [51]. *Household (family) structure* describes the relationship between parents/adults living in the household in 3 categories – two married parents, two unmarried parents and single parent households.

### Analysis plan

Initial descriptive analysis included all children 0–5 for whom breastfeeding and parental nativity data were reported. Then subsequent bivariate and multivariate analyses were restricted to children of FBNH mothers and excluded children who had U.S.-born moms and those who were of Hispanic or Latino origin (Fig. 1).

Frequencies for each variable were calculated across the 3 foreign-birth and ethnicity groups (FBNH, FBH and U.S.-born). National prevalence estimates were obtained for BFI, EBF at 6 months, BF duration ≥ 12 months and optimal BF. Estimates were also stratified by key socioeconomic factors and covariates, and both chi-square and log-binomial regression methods were used to describe overall and sub-group differences for each outcome-exposure combination at the population level.

Multivariable log-binomial regression models were then used to assess crude and adjusted associations between the socioeconomic factors of interest- CS support, WIC participation, MES- and the breastfeeding outcomes- BFI, EBF, BF duration, and optimal BF, among FBNH mothers. We checked for collinearity and removed variables that were highly correlated with any of the key exposures. We also conducted sensitivity analyses to explore differences by MES and to see how alternative definitions of EBF and BF duration might affect sample size and results. All analyses were conducted in SAS-callable SUDAAN (version 9.4) [57], using appropriate stratification and weighting measures.

## Results

We examined breastfeeding practices among mothers of children 0–5 in the NSCH. All sociodemographic characteristics and BF practices of the three groups of mothers (FBNH, FBH and U.S.-born) are detailed in Table 1. 87.7% of FBNH mothers initiated breastfeeding, 28.2% EBF for 6 months, 52.9% breastfed for ≥ 12 months, and 24.1% reported meeting all 3 breastfeeding practices (optimal BF). The median duration of breastfeeding among those who had stopped breastfeeding was 9.7 months (IQR: 5.1–14.9, *p* < 0.001) and 12% of FBNH mothers breastfed for the first 2 years.

**Table 1:** Demographic characteristics and breastfeeding practices of mothers of U.S. children 0-5y

	FBNH mothers (*N* = 3817)	FBH mothers (*N* = 1677)	U.S.-born mothers (*N* = 31721)	*P*-values ^c^
	Weighted % (SE)			
Participant characteristics				
Mothers age, years (mean, SE)^a^	36.2 (0.2)	34.7 (0.3)	34 (0.1)	< 0.001
Mother’s education				
High school or less	16.2 (1.6)	50.3 (2.3)	19.3 (0.6)	< 0.001
Some college or technical school	11.7 (1.1)	15.8 (1.4)	18.9 (0.5)	
College degree or higher	72.2 (1.8)	33.9 (2.1)	61.8 (0.6)	
Family structure				
Two parents, married	81.7 (1.4)	64.0 (2.3)	73.2 (0.6)	< 0.001
Two parents, not married	6.4 (1.0)	16.1 (1.6)	9.6 (0.4)	
Single parent	11.9 (1.2)	19.9 (2.2)	17.1 (0.5)	
Household poverty Level				
Very low income (≤ 99% of poverty level)	15.2 (1.2)	36.9 (2.4)	15 (0.5)	< 0.001
Low income (100%-199% of poverty level)	18.0 (1.3)	25.8 (2.1)	17.3 (0.5)	
Moderate income (200%-399% of poverty level)	25.8 (1.6)	21.6 (1.7)	29.2 (0.5)	
High income (≥ 400% of poverty level)	41.0 (1.5)	15.7 (1.5)	38.3 (0.5)	
Race of selected child				
White (non-Hispanic)	29.4 (1.6)		60.0 (0.6)	N/A
Black (non-Hispanic)	22.8 (1.6)		9.3 (0.4)	
Asian (non-Hispanic)	31.6 (1.3)		1.2 (0.1)	
Other/Multiracial (non-Hispanic)	16.1 (1.1)		9.2 (0.3)	
Hispanic		100	20.3 (0.6)	
Other children 0–5 in the household				
No	60.2 (1.5)	64.6 (2.3)	50.9 (0.6)	< 0.001
Yes	39.8 (1.5)	35.4 (2.3)	49.1 (0.6)	
Caregiver employment				
≥ 1 parent employed Full-time	90.0 (1.0)	83.2 (1.8)	91.0 (0.4)	< 0.001
≥ 1 parent employed Part-time	5.1 (0.6)	8.6 (1.1)	4.0 (0.3)	
Parent unemployed/working without pay	4.9 (0.9)	8.2 (1.6)	5.0 (0.3)	
Key exposure variables				
Maternal Employment status^b^				
Full-time	39.1 (1.9)	25.7 (2.8)	46.6 (0.8)	< 0.001
Part-time	15.0 (1.3)	13.6 (1.9)	16.5 (0.6)	
Unemployed	45.9 (2.0)	60.7 (3.2)	36.9 (0.9)	
Community & social support				
Low or no CS support	12.1 (1.0)	16.2 (1.6)	8.2 (0.4)	< 0.001
Moderate CS support	10.4 (1.0)	16.2 (2.1)	7.6 (0.3)	
High CS support	77.4 (1.3)	67.6 (2.3)	84.2 (0.4)	
WIC support				
No	80.1 (1.5)	56.7 (2.4)	82.3 (0.5)	< 0.001
Yes	19.9 (1.5)	43.3 (2.4)	17.7 (0.5)	
Infant feeding practices				
BF initiation	87.7 (1.0)	84.2 (1.7)	83.1 (0.5)	0.01
EBF for 6 months	28.2 (1.4)	31.0 (2.4)	29.7 (0.5)	0.56
Mixed feeding for 6 months	42.7 (1.7)	28.6 (2.1)	28.4 (0.5)	< 0.001
BF duration, months (median, SE)^a^	9.7 (5.1–14.9)	7.1 (3.1–13.8)	8.1 (2.9–12.8)	< 0.001
BF duration (binary)				
BF < 12 months	47.1 (1.7)	52.4 (2.7)	52.9 (0.7)	0.04
BF ≥ 12 months	52.9 (1.7)	47.6 (2.7)	47.1 (0.7)	
Optimal BF (all 3 recommended BF practices) − 12-month cutoff for BF duration				
No (sub-optimal)	75.9 (1.4)	71.2 (2.4)	71.8 (0.6)	0.12
Yes (optimal)	24.1 (1.3)	28.8 (2.4)	28.2 (0.6)	
Age at formula introduction (median, IQR)^a^	0 (0-3.1)	0 (0-2.7)	0 (0-2.7)	0.28
Age at complementary feeding (median, IQR)^a^	5.4 (4.2-6.0)	5.5 (4.8–6.3)	5.4 (4.2–6.5)	0.3

Prevalence of BF initiation was calculated among children 0-5 years, EBF at 6 months among children 6 mo-5years, and BF duration (binary) among children 1-5 years

*Abbreviations*: *BF-* breastfeeding, *CS* support – community and social support, *EBF-* exclusive breastfeeding

^a^Mean (SE) are reported for normally distributed or symmetrical continuous variables, while median and interquartile range are reported for the skewed age at formula introduction variable

^b^Details of analytic sample 2 used for maternal employment status are included in additional file 1a. The demographic characteristics of those who reported/were missing MES data are included

^c^All p-values are based on chi-square analyses, regression methods (for comparison of means) and Kruskal-Wallis (for comparing medians)

### Comparison of breastfeeding practices by foreign-birth & ethnicity

**Fig. 2:**
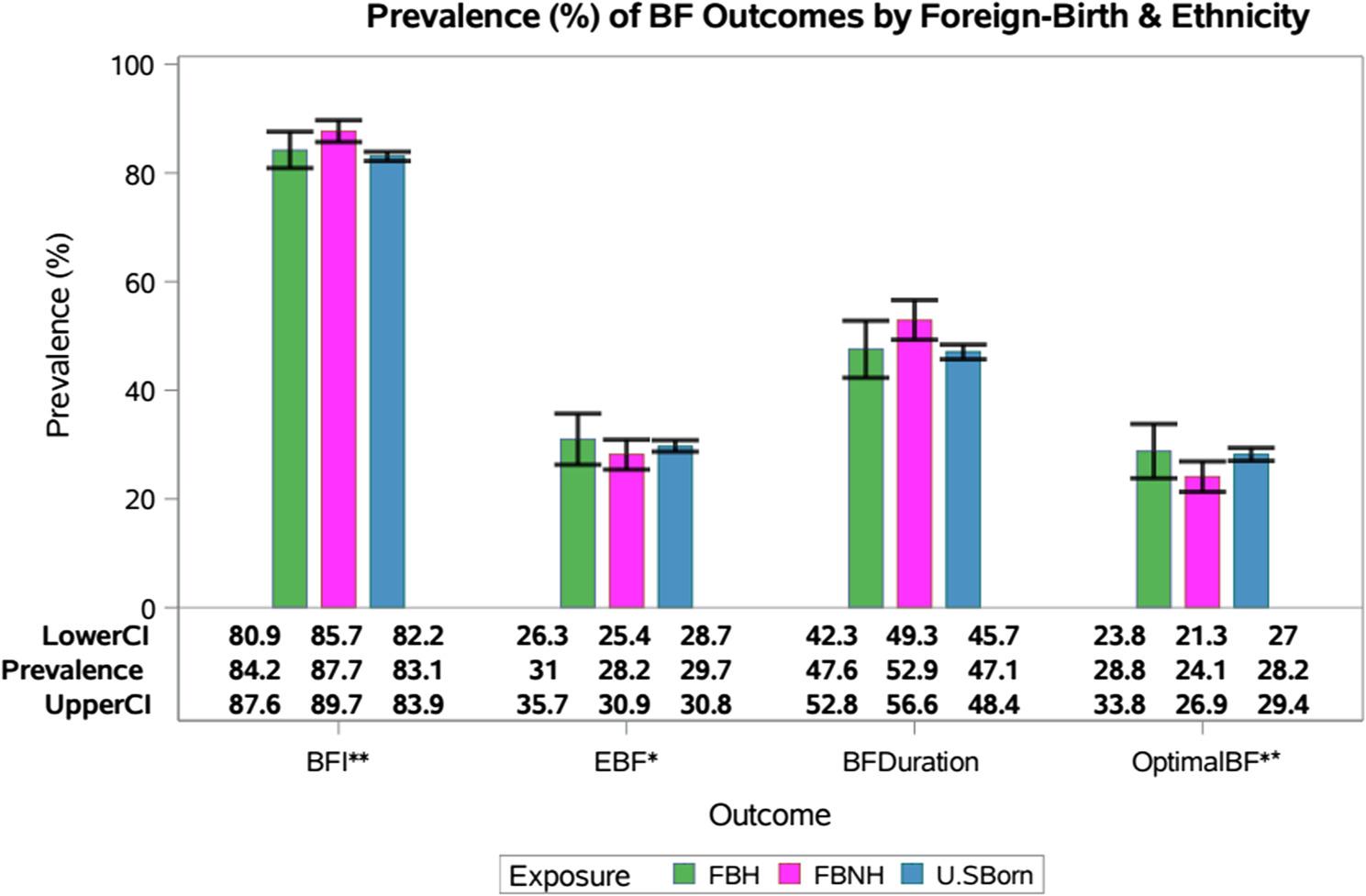
Prevalence of breastfeeding (BF) practices by maternal foreign-birth and ethnicity status Prevalence of breastfeeding (BF) practices by maternal foreign-birth and ethnicity status, National Survey of Children’s Health, United States, 2022–2023. Compared to foreign-born non-Hispanic mothers (*n*=3817), U.S.-born mothers (n=31,721) had lower prevalence of BF initiation and higher optimal BF, while foreign-born Hispanic (*n*=1677) had higher prevalence of EBF at 6 months and optimal BF. Error bars represent confidence intervals. **p<0.05 in unadjusted and adjusted models *p<0.05 in adjusted models

Overall models indicate a higher prevalence of BF initiation (*p* = 0.04) and lower prevalence of EBF for 6 months (*p* = 0.02) and optimal breastfeeding (*p* = 0.01) among FBNH relative to the other groups. Compared to U.S.-born mothers, FBNH mothers had higher rates of BF initiation (87.7% vs. 83.1%, PR = 0.95; 95% CI: 0.92–0.97) and BF for ≥ 12 months (52.9% vs. 47.1%, PR = 0.89; 95% CI: 0.82–0.96), but similar rates of EBF at 6 months (28.2% vs. 29.7%, PR = 1.06; 95% CI: 0.95–1.17) and lower rates of all 3 BF practices (24.1% vs. 28.2%, PR = 1.17; 95% CI: 1.03–1.33). Compared to FBH mothers, FBNH mothers reported comparable prevalence of BF initiation (87.7% vs. 84.2%, PR = 0.96; 95% CI: 0.92–1.01), EBF for 6 months (28.2% vs. 31%, PR = 1.10; 95% CI: 0.92–1.31), BF for ≥ 12 months (52.9% vs. 47.6%, PR = 0.90; 95% CI: 0.79–1.02) and optimal BF (24.1% vs. 28.8%, PR = 1.19; 95% CI: 0.97–1.47). Once we adjusted for other exposures and covariates of interest, prevalence of BF initiation (aPR = 0.95; 95% CI: 0.91–0.99) remained significantly lower while prevalence of optimal BF was higher (aPR = 1.23; 95% CI: 1.01–1.49) among U.S.-born mothers, compared to FBNH mothers. FBH mothers had significantly higher prevalence of EBF at 6 months (aPR = 1.50; 95% CI:1.14–1.97), and overall optimal BF (aPR = 1.69; 95% CI:1.22–2.35), compared to FBNH mothers. Prevalences for each BF practice by nativity & ethnicity group are presented in Fig. 2.

*p*

### FBNH breastfeeding practices by SES factors

**Fig. 3:**
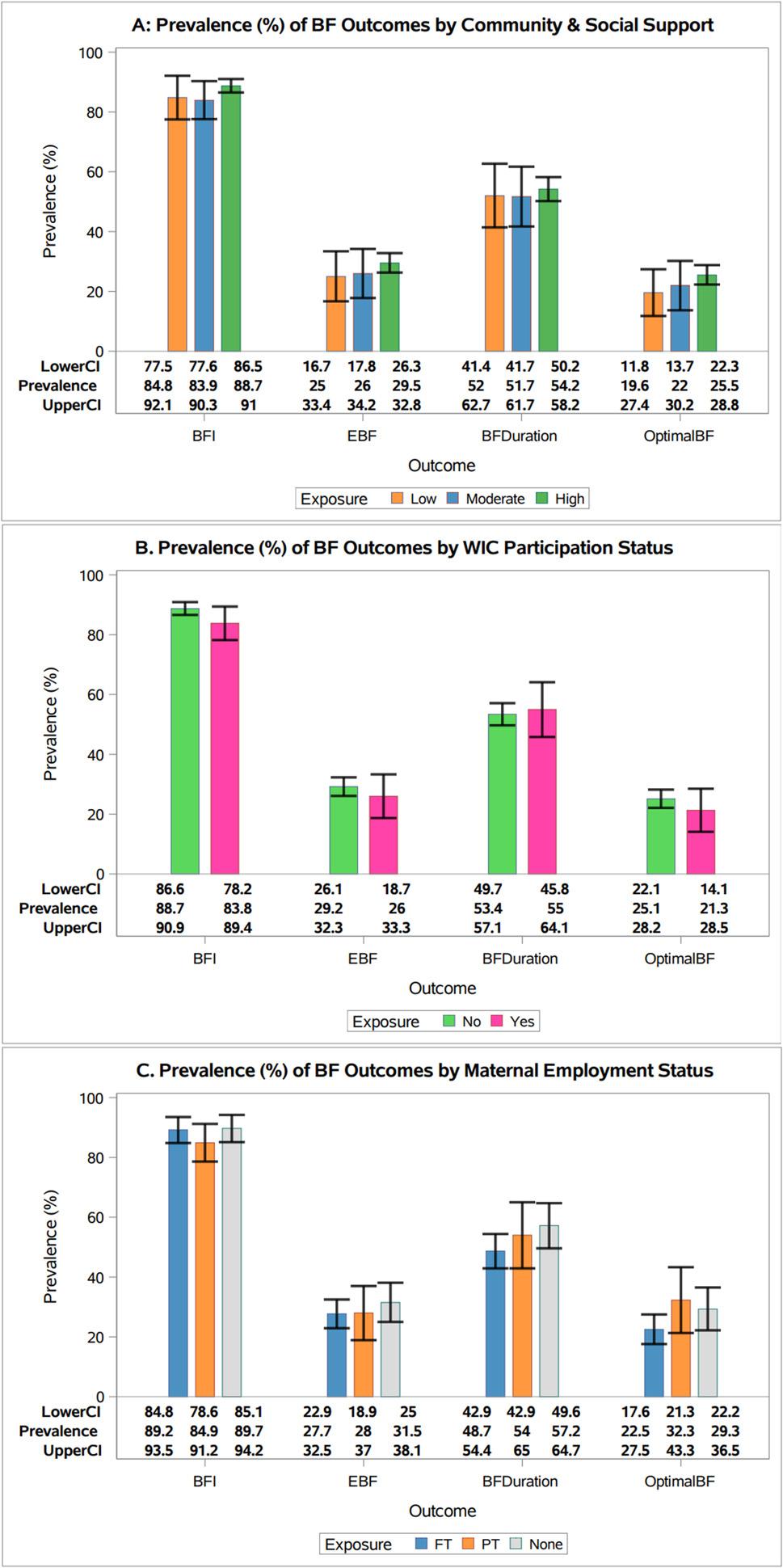
Prevalence of breastfeeding practices by community & social support, WIC participation and maternal employment status **A**. Prevalence of breastfeeding (BF) practices by level of community and social support, National Survey of Children’s Health, United States, 2022–2023 (*n*=3743). This figure displays prevalences for each breastfeeding outcome among those who reported low, moderate and high levels of community and social support. Error bars represent confidence intervals **B**. Prevalence of breastfeeding (BF) practices by WIC participation status, National Survey of Children’s Health, United States, 2022–2023 (*n*=3743). Prevalence of BF initiation, EBF for 6 months, BF ≥ 12 months, and optimal BF among those who received/did not receive WIC benefits are shown. Error bars represent confidence intervals **C**. Prevalence of breastfeeding (BF) practices by maternal employment status, National Survey of Children’s Health, United States, 2022–2023 (*n*=1808). Prevalence estimates for BF initiation, EBF for 6 months, BF ≥ 12 months, and optimal BF among mothers with full-time, part-time and no employment are shown. Error bars represent confidence intervals

FBNH mothers with high CS support scores have higher rates of EBF for 6 months (29.5% vs. 25% vs. 26%, *p* = 0.51) and optimal breastfeeding practices compared to mothers with low or moderate support (23.3% vs. 16.9% vs. 18.3%, *p* = 0.22), though the differences were not significantly different (Fig. 3A). Similarly, we did not find statistically significant differences in BF practices based on WIC participation or MES (Fig. 3B and C)

**Fig. 4:**
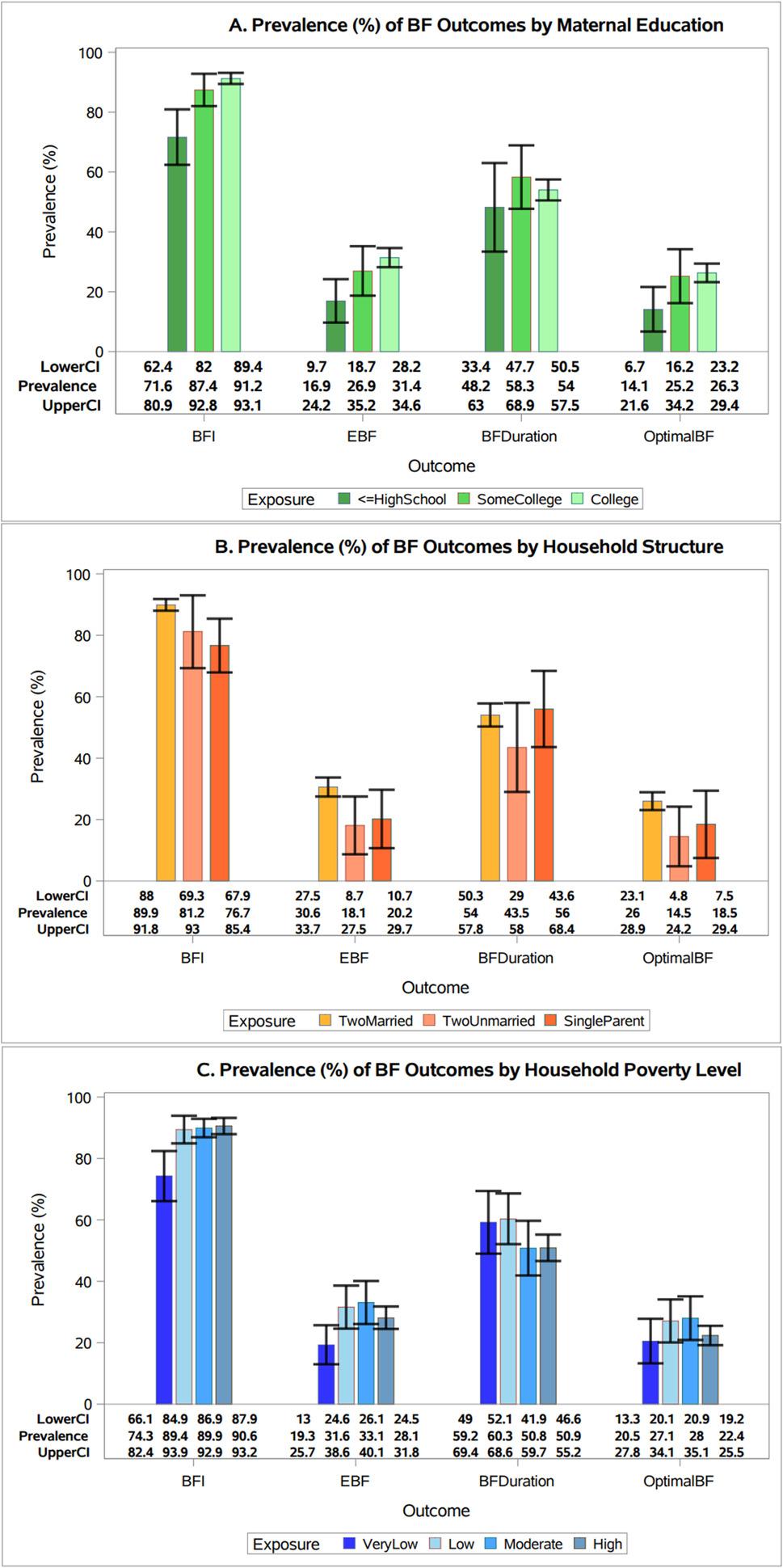
Prevalence of breastfeeding practices by maternal education, household structure and household poverty level **A**. Prevalence of breastfeeding (BF) practices by mothers’ level of education, National Survey of Children’s Health, United States, 2022–2023 (*n*=3743). Prevalence of BF initiation and EBF for 6 months was significantly higher among those with a ≥ college degree compared to the lowest education category. Error bars represent confidence intervals **B**. Prevalence of breastfeeding (BF) practices by household structure, National Survey of Children’s Health, United States, 2022–2023 (*n*=3743). The chart shows significantly higher prevalence of BF initiation and EBF for 6 months among married mothers compared to single mothers. Error bars represent confidence intervals **C**. Prevalence of breastfeeding (BF) practices by household poverty level, National Survey of Children’s Health, United States, 2022–2023 (*n*=3743). Prevalence of BF initiation and EBF for 6 months were significantly lower among mothers from very low-income households compared the higher income categories. Error bars represent confidence intervals

The proportions of FBNH mothers reporting each outcome increased with higher levels of education. FBNH mothers with some college education or higher reported higher prevalence of BFI (*p* < 0.001), EBF (*p* < 0.01), and optimal BF (*p* = 0.03) compared to FBNH mothers with a high school education or less. (Fig. 4A). Married FBNH mothers reported higher prevalence of BFI (*p* = 0.001), EBF(*p* = 0.03) and optimal BF compared to unmarried FBNH mothers (household structure is a proxy for partner support) (Fig. 4B). The prevalences of BFI (*p* = 0.001) and EBF (*p* = 0.03) were significantly lower among very low income FBNH mothers when compared to those in higher income categories (Fig. 4C). We found no statistically significant associations between the BF outcomes and other covariates (see Additional Files 2–4).

Overall, there is no evidence of significant associations between CS support, WIC participation, or MES with any of the four BF outcomes, using unadjusted and adjusted log-binomial regression models (Tables 2, 3 and 4). Sensitivity analyses indicated no significant differences between key exposures and outcomes among those with or without MES, though there were differences in child’s race and family structure between both groups (see Additional File 1a). There were also no associations detected between key exposures and outcomes when we considered EBF only among mothers who initiated BF.

**Table 2:** Prevalence ratios for community/social support and key breastfeeding outcomes among FBNH mothers

	Unadjusted model		Model 1^+^	Model 2^▲^
N= 3743*	Prevalence Ratios (95% CI)		aPR (95% CI)	aPR (95% CI)
BF Initiation				
Low or no CS support (ref)				
Moderate CS support	0.99 (0.88-1.11)		0.99 (0.90-1.08)	0.95 (0.89-1.05)
High CS support	1.05 (0.96–1.14)		1.01 (0.94-1.08)	0.96 (0.87 - 1.06)
Exclusive BF for 6 mo				
Low or no CS support (ref)				
Moderate CS support	1.04 (0.66- 1.64)		1.05 (0.68–1.64)	0.86 (0.50 - 1.48)
High CS support	1.18 (0.83–1.68)		1.07 (0.76-1.50)	0.87 (0.58 - 1.31)
BF Duration (≥12 mo)				
Low or no CS support (ref)				
Moderate CS support	0.99 (0.75 – 1.31)		1.00 (0.76 - 1.32)	0.88 (0.61-1.28)
High CS support	1.04 (0.84 – 1.29)		1.06 (0.85 - 1.31)	0.99 (0.75 - 1.31)
Optimal BF (reported all 3 practices)				
Low or no CS support (ref)				
Moderate CS support	1.12 (0.65 -1.94)		1.10 (0.65- 1.86)	1.10 (0.56 - 2.14)
High CS support	1.30 (0.85–1.98)		1.20 (0.80 – 1.80)	1.20 (0.71 - 2.03)

The table shows prevalence ratios describing the association between community and social support (exposure) and breastfeeding measures (outcomes) estimated from separate log binomial regression models

*aPR* adjusted prevalence ratios

+Model 1 - models included Community and social support and adjusted for covariates (mother’s age, education status, poverty level, number of other kids 0-5, and family structure)

▲Model 2- fully adjusted model including all 3 key exposures (community and social support, WIC participation and maternal employment status) and adjusting for all covariates (listed in model 1)

*N=3743 includes children whose mothers reported data on community and social support, WIC participation and the covariates (analytic sample 1)

**Table 3:** Prevalence ratios for WIC participation and key breastfeeding outcomes among FBNH mothers

	Unadjusted model	Model 1^+^	Model 2^▲^
*N*= 3743*	Prevalence Ratios (95% CI)	aPR (95% CI)	aPR (95% CI)
BF Initiation			
WIC Participation (Yes)	0.94 (0.88 - 1.01)	0.99 (0.93 - 1.06)	0.99 (0.91 - 1.07)
Exclusive BF for 6 mo			
WIC Participation (Yes)	0.89 (0.66 - 1.20)	1.00 (0.73 - 1.38)	1.10 (0.74 - 1.63)
BF Duration (≥12 mo)			
WIC Participation (Yes)	1.03 (0.86 - 1.24)	0.91 (0.73 - 1.13)	0.85 (0.62 - 1.17)
Optimal BF (reported all 3 practices)			
WIC Participation (Yes)	0.85 (0.59 - 1.21)	0.88 (0.59- 1.32)	1.10 (0.70 - 1.72)

The table shows prevalence ratios describing the association between WIC participation (exposure) and breastfeeding measures (outcomes) estimated from separate log binomial regression models

*aPR* adjusted prevalence ratios

+Model 1 - models included WIC participation and adjusted for covariates (mother’s age, education status, poverty level, number of other kids 0-5, and family structure)

▲Model 2- fully adjusted model including all 3 key exposures (community and social support, WIC participation and maternal employment status) and adjusting for all covariates (listed in model 1)

*N=3743 includes children whose mothers reported data on community and social support, WIC participation and the covariates (analytic sample 1)

**Table 4:** Prevalence ratios for maternal employment status and key breastfeeding outcomes among FBNH mothers

	Unadjusted model		Model 1^+^	Model 2^▲^
*N*= 1808*	Prevalence Ratios (95% CI)		aPR (95% CI)	aPR (95% CI)
BF Initiation				
Full-time (ref)				
Part-time	0.95 (0.87 - 1.04)		0.96 (0.88 - 1.04)	0.96 (0.89 - 1.05)
Unemployed	1.01 (0.94 -1.08)		1.01 (0.93 - 1.10)	1.01 (0.93 - 1.10)
Exclusive BF for 6 mo				
Full-time (ref)				
Part-time	1.01 (0.70 - 1.46)		0.97 (0.67 - 1.40)	0.92 (0.65 - 1.31)
Unemployed	1.14 (0.87 - 1.49)		1.15 (0.88 - 1.49)	1.15 (0.88 - 1.49)
BF Duration (≥12 mo)				
Full-time (ref)				
Part-time	1.11 (0.88 - 1.40)		1.02 (0.81 - 1.30)	1.03 (0.81 - 1.30)
Unemployed	1.18 (0.97 - 1.42)		1.12 (0.94 - 1.34)	1.17 (0.98 - 1.39)
Optimal BF (reported all 3 practices)				
Full-time (ref)				
Part-time	1.43 (0.95 - 2.15)		1.30 (0.87 - 1.95)	1.25 (0.84 - 1.84)
Unemployed	1.30 (0.94 - 1.81)		1.27 (0.93 - 1.74)	1.28 (0.94 – 1.74)

The table shows prevalence ratios describing the association between maternal employment status (exposure) and breastfeeding measures (outcomes) estimated from separate log binomial regression models

*aPR* adjusted prevalence ratios

+Model 1 - models included MES and adjusted for covariates (mother’s age, education status, poverty level, number of other kids 0-5, and family structure)

▲Model 2- fully adjusted model including all 3 key exposures (community and social support, WIC participation and maternal employment status) and adjusting for all covariates (mother’s age, education status, poverty level, number of other kids 0-5, and family structure)

*N=1808 includes children whose mothers had reported data for employment status and the covariates (analytic sample 2)

## Discussion

This study investigated patterns of breastfeeding among foreign-born, non-Hispanic mothers of U.S. children- a growing yet understudied group in the United States [46, 47]. FBNH mothers are more likely to initiate breastfeeding yet less likely to practice EBF for 6 months compared to other groups of mothers in the U.S. The prevalence of BF initiation and breastfeeding for ≥ 12 months was higher among FBNH mothers compared to the national average [17, 31], but they have a lower prevalence of meeting all 3 recommended breastfeeding practices compared to U.S.-born and FBH mothers. This indicates that a potential barrier to breastfeeding success of foreign-born non-Hispanic mothers in the US may not be a lack of desire to breastfeed, but a question of whether they have necessary resources and are supported to EBF for 6 months [58]. We also found that most FBNH women were practicing early supplementation with formula (median = 0 months; IQR: 0-3mo) which is a documented risk factor for suboptimal BF among infants under 6 months old [59]. This could indicate a need for targeted breastfeeding information and support for mothers in the early postpartum period. Furthermore, these results are supported by previous studies which have highlighted EBF as a challenge for immigrant mothers in the U.S. even though they often report EBF in their home country [31–34].

Children of FBNH mothers who were college educated or married had the highest prevalence of EBF while those who were from very low-income households had the lowest prevalence of EBF. However, these associations, except for marital status, were attenuated in the fully adjusted models. Being married may be an indicator of having partner and family support and previous studies have found similar positive associations with breastfeeding outcomes [60–63]. One systematic review found a positive correlation between marital relationship satisfaction and breastfeeding self-efficacy [64], which has previously been described as a predictor of EBF [65–68].

We found no evidence to support an association between CS support, WIC participation and MES and the targeted breastfeeding practices among FBNH mothers in the US. Though our analysis picked up signals that there could be some association between the 3 exposures and the various BF outcomes, we were limited in our ability to detect meaningful differences since most of our sample had high CS support, were non-WIC participants, college educated and from moderate to high income households where at least one of the adults reported full-time employment [69]. Education and poverty level are also structural determinants, but we did not consider them as key exposures because they are not as readily addressable in the short or medium-term.

A 2025 study explored exclusive and any breastfeeding through 6 months, disaggregated by foreign-birth and ethnicity status among women in the U.S [31]. Similar to our approach, they compared breastfeeding practices among U.S.-born and foreign-born women using the 2021–2022 NSCH datasets, but they did so for 6 groups disaggregated by ethnicity (non-Hispanic white, non-Hispanic Black and Hispanic). They studied outcomes only for the first 6 months of life, which makes it hard to compare all our results, but we found similar prevalence of breastfeeding initiation and EBF at 6 months with their FBH sample. Considering practical constraints for addressing breastfeeding among every sub-population within our highly heterogenous target group, we wanted to first assess common determinants of breastfeeding among FBNH mothers that could be leveraged to design targeted interventions. Our focus on FBNH mothers addresses a gap in the literature for this growing yet understudied population of mothers and contributes to the understanding of key factors that may influence their breastfeeding.

The main limitations of this study include a lack of data on parental ethnicity, acculturation status, prenatal care and context around WIC participation. Lack of parental race, ethnicity or country/region of origin measures limited our ability to explore breastfeeding practices by maternal race/region of origin or directly assess the ethnicity of the foreign-born mothers, which presents a risk of misclassification that could bias our findings. However, like previous studies [31], we leveraged child ethnicity as a proxy, which is unlikely to impact key findings. The idea is that a child with a Hispanic foreign-born mother is likely to be defined as being of “Hispanic origins” and vice versa. Also, NSCH did not collect data on the length of U.S. stay among foreign-born parents, which precluded our ability to assess acculturation status- a well-established predictor of breastfeeding practices among foreign-born mothers in the U.S [31, 70–75]. Though access to prenatal care, specifically prenatal breastfeeding education, is generally associated with improved breastfeeding outcomes [76, 77], studies assessing this relationship among immigrant populations have had mixed findings [78]. The lack of prenatal care data in the NSCH prevented direct exploration of any relationships between prenatal care and key breastfeeding outcomes among FBNH mothers. Furthermore, WIC participation was assessed from participants’ response to whether they received WIC benefits in the last 12 months, but NSCH does not ask about WIC eligibility or types of services received, so we do not have context on which type of benefits were received or how frequently, which posed a limitation in our analysis. Though we did not have data to determine the group eligible for WIC, we adjusted for poverty level which is an important criterion for WIC eligibility.

Since we used a sample of children 0–5 years old, there is a risk of recall bias especially among mothers with older children. Also, NSCH is not designed to be representative or sensitive to the experiences of FBNH mothers as they were not the target of data collection, which may have affected our group sample sizes and ability to detect clear associations. The FBNH women who responded to the survey are highly educated and moderate-to-high income, and we do not have information on those who might have declined to participate or were missing data, which could potentially introduce bias. However, we compared education and marital status among our sample to FBNH mothers in CDC Wonder birth data and found that they were also highly educated (61% had at least an associate’s degree) and mostly married (69%) [79]. To understand why FBNH mothers who have high interest in breastfeeding and do breastfeed are not able to practice EBF in the U.S., compared to their home country, we need surveys that are designed to assess these relationships and are representative by foreign-birth and ethnicity status.

Furthermore, more qualitative research is needed to gain an in-depth understanding of the complex determinants of optimal breastfeeding practices among FBNH mothers and how their region of origin, ethnicity and contexts influence infant feeding choices. Considering that foreign-born women have high parity [80], future studies should unpack the relationship between having multiple young children and optimal breastfeeding practices among FBNH mothers. Also, existing breastfeeding resources are often text-based and mostly available in English or Spanish, which may pose a limitation, especially for the FBNH mothers that are not literate in either language. Therefore, more culturally appropriate resources may be needed to promote equitable access to lactation-supportive environments and adequately support people of all origins to practice EBF for the first 6 months and breastfeed successfully [54].

## Conclusion

Foreign-born non-Hispanic mothers report a high prevalence of breastfeeding initiation and breastfeeding for the first 12 months of life, but do not follow guidelines on exclusive breastfeeding for 6 months. Compared to U.S.-born mothers, they are more likely to initiate breastfeeding, but less likely to practice EBF for 6 months and achieve all three recommended practices compared to FBH. Though we did not find evidence to conclude about associations between our main socioeconomic variables of interest and the optimal breastfeeding practices, this study highlights a need for further exploration of modifiable predictors of EBF among FBNH mothers. More research is needed to understand why FBNH mothers struggle to practice EBF in the U.S. and design context and culturally appropriate programs to maintain a culture of optimal breastfeeding. Investing in education and support systems to promote EBF for 6 months among FBNH mothers could help move the needle in progress towards the Healthy People 2030 BF goals and improve health outcomes among FBNH mothers and their children. 

## Supplementary Information

Additional File 1 A. Demographic Characteristics and BF Practices among Participants with/without Maternal Employment Status Data (*n* = 3817). National Survey of Children’s Health, 2022–2023. Word file.

Additional File 1B. Comparison of Demographic Characteristics and BF Practices among Children of FBNH Mothers (*n* = 3817) Using Two Definitions of BF Duration. National Survey of Children’s Health, 2022–2023. Word file.

Additional Files 2–4. Clustered bar charts displaying prevalence of breastfeeding practices by mothers’ age, child race and presence of multiple children 0–5 in the household; National Survey of Children’s Health, 2022–2023 (*n* = 3743). PDF file.

## Data Availability

The National Survey of Children’s Health datasets analyzed during the current study are available on the Child & Adolescent Health Measurement Initiative (CAHMI) website at https://www.childhealthdata.org/learn-about-the-nsch/NSCH

## Abbreviations

BF: Breastfeeding
CS support: Community and social support
EBF: Exclusive breastfeeding
FBH: Foreign-born Hispanic
FBNH: Foreign-born non-Hispanic
MES: Maternal Employment Status
NSCH: National Survey of Children’s Health
WIC: Special Supplemental Nutrition Program for Women, Infants, and Children
