# Supplementary material for: Breastfeeding practices among foreign-born non-Hispanic mothers of children in the United States – a cross-sectional study of nationwide multi-year data
Source: BMC Pregnancy Childbirth. 2026 Apr 17;26:579. doi: 10.1186/s12884-026-09082-5 (PMC13217829; doi:10.1186/s12884-026-09082-5)
Supplement: Supplementary file 1 — Additional File 1 A. Demographic Characteristics and BF Practices among Participants with/without Maternal Employment Status Data (n = 3817). National Survey of Children’s Health, 2022–2023. Word file. [file 12884_2026_9082_MOESM1_ESM.docx]

**Additional File 1A**: Demographic Characteristics and BF Practices among Participants with/without Maternal Employment Status Data

|  | **MES (N= 1810)** | | **Missing MES (N= 2007)** | | | **p-value** |
| --- | --- | --- | --- | --- | --- | --- |
|  | N | weighted % (SE) | | N | weighted % (SE) |  |
| Participant characteristics | | | | | |  |
| *Mothers age, years* (mean, SE)^a^ | 36.4 (0.3) | - | | 36.3 (0.3) | - | 0.58 |
| *Mother’s education* |  | | | | |  |
| High school or less | 108 | 13.8 (1.9) | | 196 | 18.0 (2.4) | 0.25 |
| Some college or technical school | 177 | 10.9 (1.7) | | 225 | 12.3 (1.7) |  |
| College degree or higher | 1523 | 75.3 (2.3) | | 1586 | 69.7 (2.6) |  |
| *Family structure** |  | | | | |  |
| Two parents, married | 1671 | 89.9 (1.8) | | 1608 | 75.0 (2.0) | <0.001 |
| Two parents, not married | 92 | 5.8 (1.2) | | 86 | 6.8 (1.5) |  |
| Single parent | 45 | 4.3 (1.5) | | 308 | 18.2 (1.8) |  |
| *Household poverty Level* |  | | | | |  |
| Very low income (≤99% of poverty level) | 149 | 12.9 (1.9) | | 258 | 17.0 (1.6) | 0.34 |
| Low income (100%-199% of poverty level) | 246 | 18.7 (2.1) | | 302 | 17.4 (1.6) |  |
| Moderate income (200%-399% of poverty level) | 474 | 24.3 (1.6) | | 507 | 27 (2.6) |  |
| High income (≥400% of poverty level) | 939 | 44.1 (2.1) | | 940 | 38.6 (2.2) |  |
| *Race of Selected Child** |  | | | | |  |
| White (Non-Hispanic) | 543 | 29.5 (2.1) | | 617 | 29.4 (2.4) | 0.02 |
| Black (Non-Hispanic) | 171 | 18.9 (2.2) | | 246 | 26.1 (2.3) |  |
| Asian (Non-Hispanic) | 768 | 34.1 (2.0) | | 811 | 29.6 (1.8) |  |
| Other/Multiracial (Non-Hispanic) | 326 | 17.5 (1.5) | | 333 | 14.9 (1.5) |  |
| *Other children 0-5 in the household* |  | | | | |  |
| 0 | 1231 | 60.7 (1.9) | | 1378 | 59.7 (2.3) | 0.99 |
| 1 or more | 577 | 39.3 (1.9) | | 620 | 40.3 (2.3) |  |
| *Caregiver employment* |  | | | | |  |
| ≥1 parent employed Full-time | 1721 | 92.9 (1.6) | | 1768 | 87.6 (1.4) | 0.06 |
| ≥1 parent employed Part-time | 54 | 3.1 (0.5) | | 129 | 6.9 (1.0) |  |
| Parent unemployed/working without pay | 33 | 4.0 (1.6) | | 90 | 5.5 (1.0) |  |
| Key exposure variables | | | | | |  |
| *Maternal Employment status* |  | | | | |  |
| Full-time | 830 | 39.2 (1.9) | |  |  | N/A |
| Part-time | 296 | 15.0 (1.3) | |  |  |  |
| Unemployed | 682 | 45.8 (2.0) | |  |  |  |
| *Community & Social Support* |  | | | | |  |
| Low or no CS support | 160 | 10.5 (1.4) | | 218 | 13.4 (1.5) | 0.14 |
| Moderate CS support | 156 | 8.9 (1.2) | | 213 | 11.6 (1.3) |  |
| High CS support | 1482 | 80.5 (1.8) | | 1549 | 74.9 (1.8) |  |
| *WIC participation* | 203 | 18.9 (2.1) | | 279 | 20.7 (1.9) | 0.65 |
| Infant feeding Practices | | | | | |  |
| *BF initiation* | 1654 | 88.8 (1.5) | | 1755 | 86.8 (1.4) | 0.50 |
| *EBF for 6 months* | 535 | 29.5 (1.9) | | 529 | 27.1 (2.0) | 0.41 |
| *Mixed feeding for 6 months* | 731 | 42.4 (2.3) | | 798 | 42.9 (2.4) | 0.88 |
| *BF duration*, months (median, IQR)^a^ | 8.9 (4.6 – 14.6) | - | | 10.1 (5.2-15.3) | - | 0.24 |
| *BF Duration* |  | | | | |  |
| BF for < 12 months | 750 | 56.0 (2.1) | | 761 | 51.9 (2.5) | 0.26 |
| BF for ≥ 12 months | 505 | 31.5 (1.9) | | 549 | 35.5 (2.3) |  |
| *BF for ≥ 24 months* | 180 | 12.5 (1.4) | | 202 | 12.7 (1.6) |  |
| *Optimal BF (all 3 recommended BF practices)-* 12-month cutoff for BF duration |  | | | | |  |
| No (sub-optimal) | 1082 | 75.5 (1.8) | | 1164 | 80.4 (1.8) | 0.08 |
| Yes (optimal) | 340 | 24.5 (1.8) | | 333 | 19.6 (1.8) |  |
| *Age at formula introduction* (median, IQR)^a^ | 0 (0-3.3) | - | | 0 (0-3.0) | - | 0.29 |
| *Age at complementary feeding* (median, IQR)^a^ | 5.4 (4.2 – 6.0) | - | | 5.4 (4.1 -6.3) | - | 0.92 |

*^a^Mean (SE) are reported for normally distributed or symmetrical continuous variables, while median and interquartile range are reported for variables with a skewed distribution.*

**Indicates variables that differ significantly between mothers with or without employment status data based on sensitivity analyses.*

*Abbreviations: BF- breastfeeding; CS support – community and social support; EBF- exclusive breastfeeding*
