# Supplementary material for: Breastfeeding practices among foreign-born non-Hispanic mothers of children in the United States – a cross-sectional study of nationwide multi-year data
Source: BMC Pregnancy Childbirth. 2026 Apr 17;26:579. doi: 10.1186/s12884-026-09082-5 (PMC13217829; doi:10.1186/s12884-026-09082-5)
Supplement: Supplementary file 2 — Additional File 1B. Comparison of Demographic Characteristics and BF Practices among Children of FBNH Mothers (n = 3817) Using Two Definitions of BF Duration. National Survey of Children’s Health, 2022–2023. Word file. [file 12884_2026_9082_MOESM2_ESM.docx]

**Table 1b.1**: Comparison of Demographic Characteristics and BF Practices among Children of FBNH Mothers with Suboptimal BF Duration (< 12months) Using Two Definitions

|  | **BF Duration 1- Children 0-5y (N=2949)** | | **BF Duration 2- Children 1-5y (N= 3060)** | |
| --- | --- | --- | --- | --- |
|  | BF Duration < 12mo | | | |
|  | N | weighted % (SE) ^a^ | N | weighted %( SE) ^a^ |
| Participant characteristics | | | | |
| *Mothers age, years* (mean, SE)^b^ | 36.1 (0.3) |  | 36.5 (0.3) |  |
| *Mother’s education* |  | | | |
| High school or less | 107 | 8.8 (1.9) | 100 | 8.1 (1.9) |
| Some college or technical school | 165 | 5.3 (0.6) | 154 | 4.7 (0.6) |
| College degree or higher | 1241 | 39.7 (1.8) | 1163 | 34.3 (1.7) |
| *Family structure** |  | | | |
| Two parents, married | 1281 | 44.6 (1.9) | 1198 | 38.6 (1.8) |
| Two parents, not married | 82 | 3.6 (0.7) | 77 | 3.3 (0.6) |
| Single parent | 147 | 5.5 (0.8) | 139 | 5.1 (0.8) |
| *Household poverty Level* |  | | | |
| Very low income (≤99% of poverty level) | 139 | 6.2 (0.8) | 130 | 5.7 (0.8) |
| Low income (100%-199% of poverty level) | 213 | 9.8 (1.3) | 192 | 8.0 (1.1) |
| Moderate income (200%-399% of poverty level) | 382 | 14.5 (1.9) | 359 | 13.0 (1.8) |
| High income (≥400% of poverty level) | 779 | 23.3 (1.4) | 736 | 20.4 (1.3) |
| *Race of Selected Child** |  | | | |
| White (Non-Hispanic) | 451 | 17.0 (1.8) | 430 | 15.6 (1.8) |
| Black (Non-Hispanic) | 141 | 9.4 (1.3) | 132 | 8.7 (1.2) |
| Asian (Non-Hispanic) | 641 | 17.5 (1.3) | 598 | 14.5 (1.0) |
| Other/Multiracial (Non-Hispanic) | 280 | 9.8 (1.1) | 257 | 8.3 (1.0) |
| *Other children 0-5 in the household* |  | | | |
| 0 | 1050 | 33.0 (1.9) | 1006 | 29.1 (1.8) |
| 1 or more | 463 | 20.8 (1.6) | 411 | 17.9 (1.5) |
| *Caregiver employment* |  | | | |
| ≥1 parent employed Full-time | 1390 | 49.7 (1.8) | 1303 | 43.5 (1.8) |
| ≥1 parent employed Part-time | 69 | 2.3 (0.5) | 66 | 2.2 (0.5) |
| Parent unemployed/working without pay | 42 | 1.4 (0.4) | 37 | 1.1 (0.3) |
| Key exposure variables | | | | |
| *Maternal Employment status* |  | | | |
| Full-time | 372 | 24.3 (1.7) | 349 | 20.9 (1.6) |
| Part-time | 112 | 7.9 (1.2) | 100 | 6.7 (1.1) |
| Unemployed | 268 | 23.8 (2.3) | 251 | 19.3 (2.1) |
| *Community & Social Support* |  | | | |
| Low or no CS support | 154 | 6.2 (0.92) | 144 | 5.6 (0.9) |
| Moderate CS support | 157 | 5.5 (0.8) | 155 | 5.3 (0.8) |
| High CS support | 1185 | 41.9 (1.9) | 1101 | 36.0 (1.8) |
| *WIC participation* | 186 | 9.7 (1.3) | 163 | 7.8 (1.1) |
| Infant feeding Practices | | | | |
| *BF initiation* | 1513 | 53.8 (1.7) | 1417 | 47.1 (1.7) |
| *EBF for 6 months* | 261 | 9.8 (1.2) | 253 | 8.9 (1.1) |
| *Mixed feeding for 6 months* | 602 | 20.7 (1.5) | 583 | 18.8 (1.4) |
| *Optimal BF (all 3 recommended BF practices)-* 12-month cutoff for BF duration |  | | | |
| No (sub-optimal) | 1489 | 53.1 (1.7) | 1416 | 47.1 (1.7) |
| Yes (optimal) | 0 | 0 | 0 | 0 |
| *Age at formula introduction* (median, IQR)^b^ | 0 (0-4.3) |  | 0(0-4.7) |  |
| *Age at complementary feeding* (mean, SE)^b^ | 5.5 (4.2 – 8.1) |  | 5.5 (4.0 – 7.8) |  |

*BF duration was defined in two ways using i)age at breastfeeding cessation (months) among all children 0-5y for whom that information was reported and ii) using age at breastfeeding cessation for children who were at least 12 months old at the time of the survey (1-5y) and children 1-5 years old who were still breastfeeding.*

*^a^Percentages document the proportion of children of FBNH mothers who report suboptimal BF Duration by each exposure level using definition 1 vs. 2. The purpose is to compare suboptimal BF among children of FBNH mothers based on the two definitions.*

*^b^Mean and standard errors are reported for normally distributed or symmetrical maternal age variable, while median and interquartile range are reported for the skewed age at formula and solids introduction variables*

*Abbreviations: BF- breastfeeding; CS support – community and social support; EBF- exclusive breastfeeding*

Table 1b.2: Comparison of prevalence ratios by nativity & ethnicity using two definitions of BF duration and optimal BF

|  | Unadjusted model |  | Adjusted Model^+^ |  |
| --- | --- | --- | --- | --- |
| *N= 37,215* | Prevalence Ratios (95% CI) | Model p-value | aPR (95% CI) | Overall model p-values |
| **BF Duration ≥ 12mo (0-5y)- definition 1^a^** |  | | | |
| FBNH (ref) | 1.00 | 0.02 | 1.00 | 0.06 |
| FBH | 0.88 (0.76 -1.03) |  | 1.35 (1.07 - 1.71) |  |
| U.S. Born | 0.88 (0.81-0.96) |  | 1.07 (0.93 - 1.23) |  |
| **BF Duration ≥ 12mo (1-5y)- definition 2^b^** |  |  |  |  |
| FBNH (ref) | 1.00 | 0.01 | 1.00 | 0.07 |
| FBH | 0.90 (0.79-1.02) |  | 1.24 (1.02-1.51) |  |
| U.S. Born | 0.89 (0.82 - 0.96) |  | 1.00(0.89 - 1.14) |  |
| **Optimal BF (0-5y)- definition 1** |  |  | | |
| FBNH (ref) | 1.00 | 0.13 | 1.00 | 0.04 |
| FBH | 1.13 (0.89 - 1.44) |  | 1.64 (1.12 -2.41) |  |
| U.S. Born | 1.15 (1.00 - 1.31) |  | 1.22 (0.99 - 1.51) |  |
| **Optimal BF (1-5y) - definition 2** |  | | | |
| FBNH (ref) | 1.00 | 0.04 | 1.00 | 0.01 |
| FBH | 1.19 (0.97-1.47) |  | 1.69 (1.22 - 2.35) |  |
| U.S. Born | 1.17 (1.03-1.33) |  | 1.23 (1.01 - 1.49) |  |

*BF duration was defined in two ways using i)age at breastfeeding cessation (months) among all children 0-5y for whom that information was reported and ii) using age at breastfeeding cessation for children who were at least 12 months old at the time of the survey (1-5y) and children 1-5 years old who were still breastfeeding. The Optimal BF variables combine the other 3 outcomes (BF initiation, EBF for 6 months, and BF duration); each variation is based on the version of BF duration used.*
