# Supplementary material for: Breastfeeding practices among foreign-born non-Hispanic mothers of children in the United States – a cross-sectional study of nationwide multi-year data
Source: BMC Pregnancy Childbirth. 2026 Apr 17;26:579. doi: 10.1186/s12884-026-09082-5 (PMC13217829; doi:10.1186/s12884-026-09082-5)

Additional File 2. Mean Age of Mothers who Reported Each Breastfeeding Practice

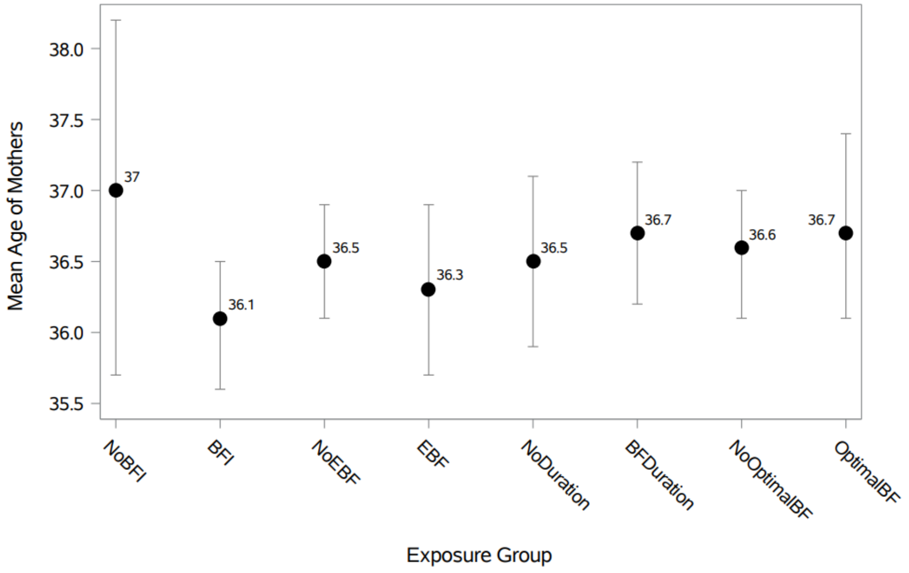

Additional File 3. Prevalence (%) of BF Outcomes by Child Race

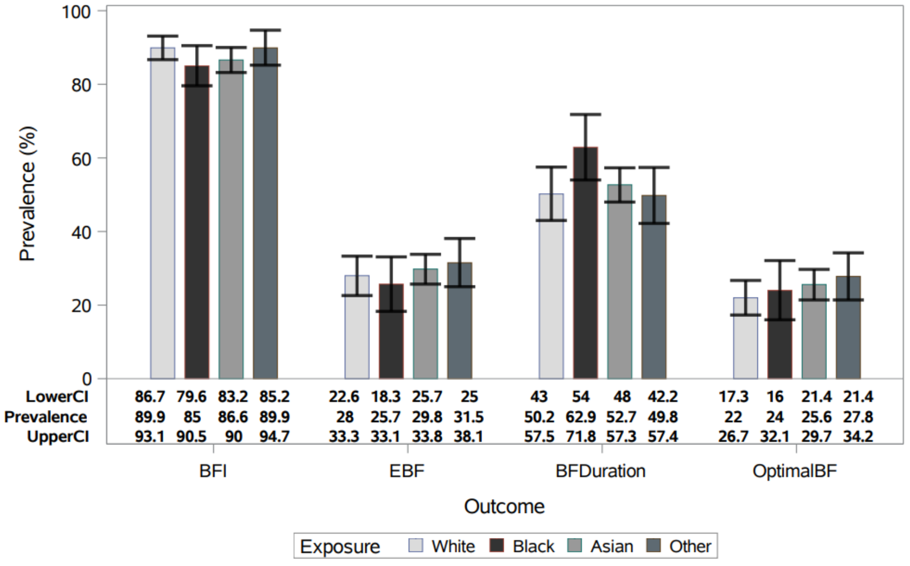

Additional File 4. Prevalence (%) of BF Outcomes by Presence of Multiple Children 0-5 in the Household

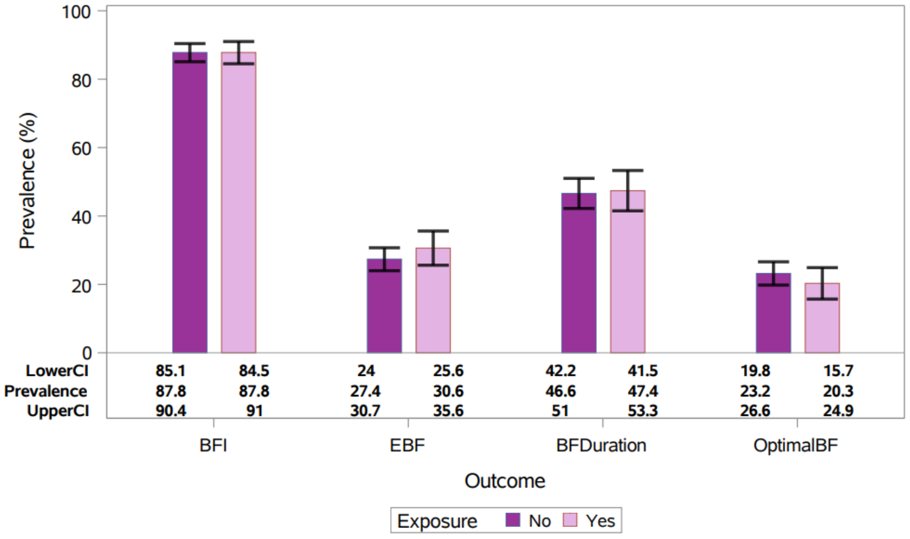

Supplement: Supplementary file 3 — Additional Files 2–4. Clustered bar charts displaying prevalence of breastfeeding practices by mothers’ age, child race and presence of multiple children 0–5 in the household; National Survey of Children’s Health, 2022–2023 (n = 3743). PDF file. [file 12884_2026_9082_MOESM3_ESM.pdf]
